# Biliary Neuroendocrine Neoplasms: Clinical Profiles, Management, and Analysis of Prognostic Factors

**DOI:** 10.3389/fonc.2019.00038

**Published:** 2019-02-05

**Authors:** Zhibo Zheng, Chuyan Chen, Binglu Li, Hongbo Liu, Liangrui Zhou, Hui Zhang, Chaoji Zheng, Xiaodong He, Wei Liu, Tao Hong, Yupei Zhao

**Affiliations:** ^1^Department of General Surgery, Peking Union Medical College Hospital, Chinese Academy of Medical Sciences, Beijing, China; ^2^School of Public Health, China Medical University, Shenyang, China; ^3^Department of Pathology, Peking Union Medical College Hospital, Chinese Academy of Medical Sciences, Beijing, China

**Keywords:** neuroendocrine neoplasm, gallbladder, biliary tract, ampulla, survival

## Abstract

Biliary neuroendocrine neoplasms (NENs) represent <1% of all NENs. The aim of this retrospective study is to present the clinical characteristics, management and prognosis profiles of 28 biliary NEN patients from a large tertiary center, and identify factors related to prognosis. Nine tumors originated from the gallbladder, two from the extrahepatic bile duct and 17 from the ampulla of Vater. One patient was classified as neuroendocrine tumor (NET) Grade 1, three patients were classified as NET Grade 2, 18 were graded neuroendocrine carcinoma (NEC) Grade 3 and six were classified as mixed adenoneuroendocrine carcinoma (MANEC). The overall survival rate and disease-free survival rate did not have statistically significant differences between tumors of different locations or different grading. Recurrence of disease correlated with poor prognosis (*p* < 0.001). Lymphovascular invasion and invasion beyond the submucosa were related to higher risk of local lymph node metastases. Multivariate analysis identified patient age (*p* = 0.021) and R0 resection margin (*p* = 0.027) as independent prognostic factors associated with overall survival. Our study included relatively large numbers of biliary tract NENs with intact follow-up information. Patients with biliary neuroendocrine tumors showed different clinical outcomes according to tumor locations and tumor grades. Achieving R0 resection is important for better prognosis.

## Introduction

Gastroenteropancreatic neuroendocrine neoplasms (GEP-NENs) are a diverse spectrum of solid tumors arising from the neuroendocrine cell system. The incidence of GEP-NENs has been increasing in recent years, possibly due to improved diagnostic techniques and increased awareness of the disease (1). Data from Surveillance, Epidemiology, and End Results (SEER) program demonstrated a development of 2.53 new cases of GEP-NENs per 100,000 people in 2012 (2). The small intestine (1.05 per 100,000 persons), the rectum (1.04 per 100,000 persons) and the pancreas (0.48 per 100,000 persons) were the most common sites of GEP-NENs (SEER 18: 2000–2012) (2).

NEN originating from the biliary system has been very rare. Modlin et al. analyzed information of 13,715 patients diagnosed of NEN and reported only 42 cases located in the gallbladder and 111 cases located in extrahepatic biliary ducts, accounting for only ~2% of the GEP-NENs (3). After that, clinical information of neuroendocrine neoplasms arising in the biliary tract has been investigated in few papers (4–9). But due to the rarity of such neoplasms, problems still exist. Whether the site of origin (e.g., gallbladder and biliary tract vs. ampulla of Vater) could influence tumor type or prognosis, and whether pathological grading system could predict tumor behavior and patient prognosis still requires further investigation. Also, the rapid update of NEN classification systems in recent years calls on for a more circumspect analysis of the histopathological pattern of NENs originating from the biliary tract.

Thus, the aim of this study was to summarize the clinicopathological features of biliary tract NENs patients from a large tertiary center, and identify factors related to prognosis.

## Materials and Methods

### Patients and Clinical Information

We performed a retrospective study of 36 patients diagnosed with biliary neuroendocrine tumors who were referred to Peking Union Medical College Hospital from 1991 to 2017. Histological assessment of tumor tissues was performed at the Pathology Department of Peking Union Medical College Hospital. The presence of a confirmed NEN histology was required to make the diagnosis. Biliary NEN was diagnosed if the primary tumor located in the gallbladder, biliary tract, or ampulla of Vater. Although NEN of the ampulla region should follow the TNM classification of small intestine NEN, previous studies included AoV NENs in comparison due to its similar clinical features with tumors of the extrahepatic biliary tract (4). Eight patients were excluded from the study because they only underwent biopsy. A final total of 28 patients with biliary NEN were identified. Patients were enrolled after providing oral consent and this study was approved by the Institutional Review Board of Peking Union Medical College Hospital (S-K597). This study was performed in accordance with the 1964 Helsinki Declaration and its later amendments ethical standards.

In this study, data collection was obtained by manual retrieval of patient records. For each patient, age, gender, the location of tumors, operation method, pathologic diagnosis, metastases status, and outcome were extracted from the medical records. Subsequent follow up was conducted at our outpatient clinic at an interval of 1–3 months in the beginning, and extended to 6–12 months if no recurrence or disease progression occurs. Patients who refused to come back for follow up were called by phone to check their present health status.

### Histology Classification

Immunohistochemical tests were performed for Ki-67 (monoclonal, clone EP5, ZSGB-BIO, Beijing, PRC), chromogranin A (monoclonal, clone EP38, ZSGB-BIO, Beijing, PRC), synaptophysin (monoclonal, clone 27G12, Leica Biosystems Newcastle Ltd, Newcastle, UK), and CD56 (monoclonal, clone CD564, Leica Biosystems Newcastle Ltd, Newcastle, UK) in all cases. All tumor samples were reviewed by two surgical pathologists specialized in neuroendocrine pathology.

Neuroendocrine tumors were classified according to WHO 2010 classification(10, 11). Grading of the tumor was based on The ENETS grading classification: NET G1 (mitotic count < 2 per 10 HPF and/or a Ki-67 index ≤ 2%); NET G2 (mitotic count 2–20 per 10 HPF and/or Ki-67 index of 3–20%); NEC G3 (mitotic count >20 per 10 HPF and/or Ki-67 index >20%). MANEC was defined as tumors that are morphologically recognizable as both exocrine and endocrine component, with one component exceeding 30%.

R0 resection margin was defined as no cancer cells seen microscopically at the resection margin. R1 resection margin was defined as microscopic positive margins.

### Statistics

Continuous variables were presented as the average ± standard deviation. Categorical variables were compared by Fisher's exact test. Continuous variables were compared by ordinary one-way ANOVA. Disease-free survival and overall survival rates were analyzed by the log-rank test. ROC analysis was used to determine cutoff values. Multiple Cox regression analysis was used to evaluate factors related to overall survival. *P* < 0.05 was considered statistically significant. Statistical analysis was performed using SPSS version 25.0 for Windows (SPSS Inc., Chicago, IL, USA) and GraphPad Prism version 7 for Windows (GraphPad Software, CA, USA).

## Results

### Patients

In the study, 28 patients from Peking Union Medical College Hospital (1991–2017) were investigated and followed up for a median of 21.5 months. The whole patient series was composed of 19 males and eight females (Table 1). The average age of disease presentation was 55.2 ± 11.2 years. According to the location, there were nine NENs originated from the gallbladder, two from the biliary tract and 17 from the ampulla of Vater (AoV). There were no cases of NEN from the intrahepatic bile duct.

**Table 1:** Characteristics of patients.

	**Gallbladder**	**Biliary tract**	**Ampulla of Vater**
	***N* = 9 (%)**	***N* = 2 (%)**	***N* = 17 (%)**
Gender (male %)	3 (33.3)	2 (100)	14 (82.4)
Median Age (year)	59	52.5	50
Abdominal discomfort (%)	3 (33.3)	0	11 (64.7)
Jaundice (%)	0	2 (100)	10 (58.8)
Fever (%)	1 (11.1)	0	2 (11.8)
Nausea-vomiting (%)	1 (11.1)	0	2 (11.8)
Anorexia (%)	1 (11.1)	0	1 (5.9)
Weight loss (%)	0	0	1 (5.9)
Weakness (%)	0	0	1 (5.9)
Hormonal symptoms (%)	0	0	0

The tumors were symptomatic in 82.1% of the patients. The most common symptom was abdominal discomfort (50%). Jaundice was observed in 64.7% of patients with AoV NEN. Other symptoms that were described include fever, nausea-vomiting, anorexia, weight loss, and weakness. No hormonal symptoms were observed. Most gallbladder NENs were incidentally diagnosed during ultrasound or radiological imaging.

### Histology

**Figure 1:**
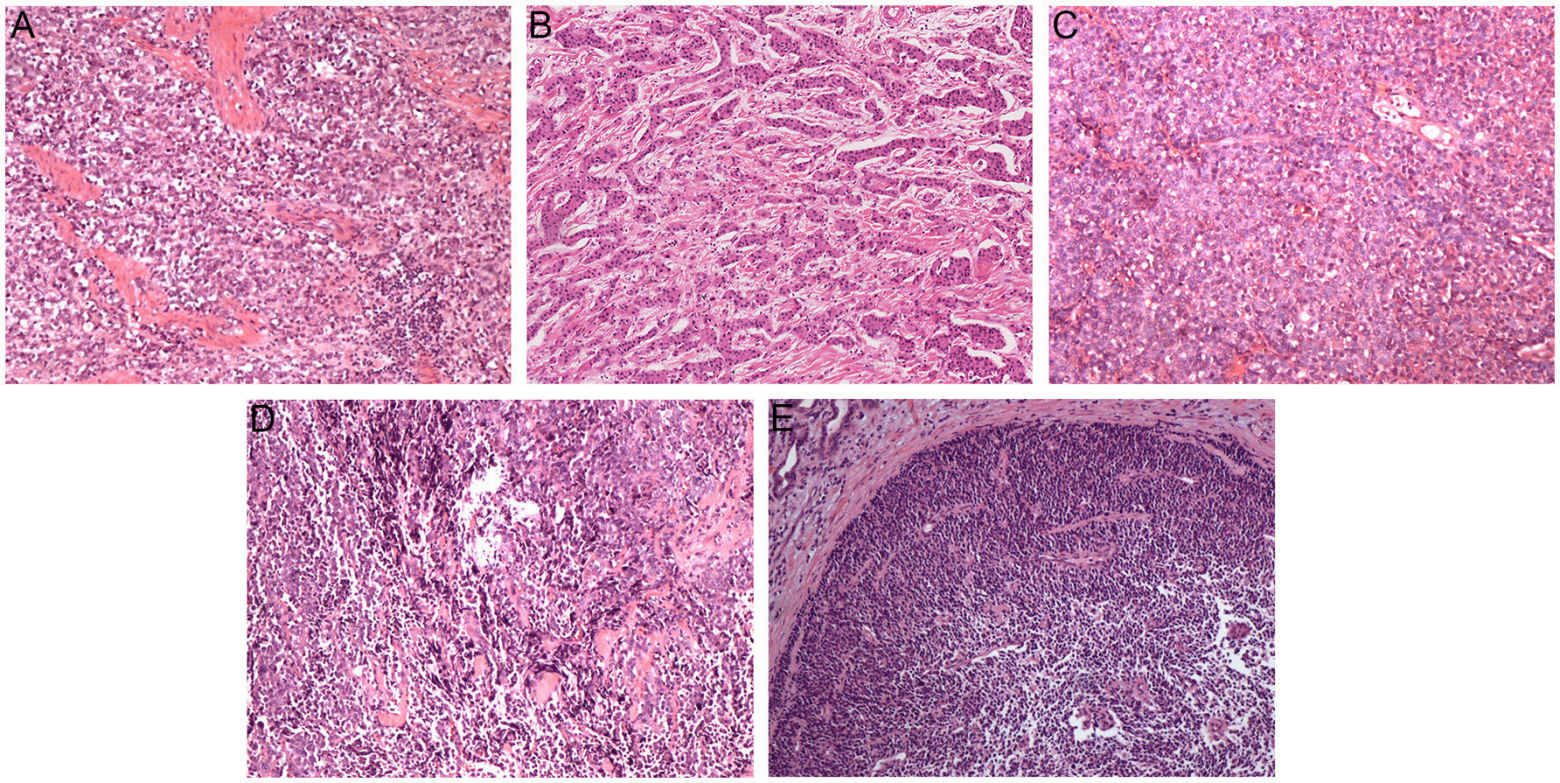
Pathological findings of biliary neuroendocrine neoplasms (hematoxylin-eosin, original magnification × 100). Tumor cells were poorly differentiated and arranged in cellular nests and sheets **(A–C)**. Neuroendocrine carcinoma cells with differentiated adenocarcinoma cells **(D,E)**. **(A)** Gallbladder neuroendocrine carcinoma; **(B)** Biliary tract neuroendocrine carcinoma; **(C)** Ampulla of Vater neuroendocrine carcinoma; **(D)** Gallbladder mixed adenoneuroendocrine carcinoma; **(E)** Ampulla of Vater mixed adenoneuroendocrine carcinoma.

Figure 1 shows pathological findings of biliary neuroendocrine neoplasms and pathohistologic features are presented in Table 2. Among the 28 patients, one patient was classified as Grade 1 (G1) NET, three as Grade 2 (G2) NET, 18 as Grade 3 (G3) neuroendocrine carcinoma (NEC), and six as mixed adenoneuroendocrine carcinoma (MANEC) based on the WHO 2010 classification (11) and ENETS grading classification (10). NENs originating from the gallbladder and biliary tract showed higher grading, as the average Ki-67 index reached 70%. NENs from the ampulla of Vater showed more diversity, but the majority were still graded G3 (70.6%). Immunohistochemical staining showed features of NENs, with no differences among the three locations.

**Table 2:** Clinicopathological features of biliary neuroendocrine tumors after curative resection according to location.

	**Gallbladder *n* = 9**	**Biliary tract *n* = 2**	**Ampulla of Vater *n* = 17**	***p*-value**
**WHO 2010 CLASSIFICATION (%)**				
G1	0	0	1 (5.9)	
G2	0	0	3 (17.6)	
G3	4 (44.4)	2 (100)	12 (70.6)	
MANEC	5 (55.6)	0	1 (5.9)	
Average Ki-67 index (%)	70 (40-85)	70	40 (1-90)	0.018
**IMMUNOHISTOCHEMICAL STAINING (%)**				
CgA	8 (88.9)	2 (100)	11 (64.7)	0.402
Syn	9 (100)	2 (100)	15 (88.2)	0.595
CD56	7 (77.8)	2 (100)	16 (94.1)	0.419
AE1/AE3	8 (88.9)	2 (100)	15 (88.2)	>0.99
Lymphovascular invasion (%)	5 (55.6)	2 (100)	10 (58.8)	0.703
**INITIAL METASTASIS (%)**				
Lymph node	4 (44.4)	2 (100)	10 (58.8)	0.501
Liver	2 (22.2)	0	1 (5.9)	0.419
Pancreas	0	0	5 (29.4)	0.193
**TNM STAGING (%)**				
0	1 (11.1)	0	1 (5.9)	
I	1 (11.1)	0	4 (23.5)	
II	7 (77.8)	0	8 (47.1)	
III	0	2 (100)	3 (17.6)	
IV	0	0	1 (5.9)	
**RESECTION MARGIN (%)**				
R0	7 (77.8)	2 (100)	17 (100)	
R1	2 (22.2)	0	0	
Median primary tumor size (cm)	2	3.95	2.5	0.203
Primary tumor size>2cm (%)	4 (44.4)	2 (100)	9 (52.9)	0.596
Recurrence (%)	6 (66.7)	2 (100)	5 (29.5)	0.068
Median DFS (mo)	5 (3–179)	37.5 (3–72)	12 (1–281)	0.478
Median OS (mo)	17 (9–179)	51.5 (7–96)	28 (1–281)	0.545
2-Year survival rate (%)	55.6	50	70.6	0.717

*WHO, World Health Organization; MANEC, mixed adenoneuroendocrine carcinoma; CgA, Chromagranin A; Syn, Synaptophysin; CD56, cluster of differentiation 56; AE1/AE3, pan cytokeratin; DFS, disease free survival; OS, overall survival*.

Lymph node metastases were observed in 16 of 28 patients, without a discrepancy between different tumor origins. Hepatic metastases were noted in two gallbladder NENs and one AoV NEN. Metastases were observed in one (100%), two (66.7%), nine (50%), and four (66.7%) patients classified G1, G2, NEC, and MANEC, respectively (Table 3). All patients with biliary tract and AoV NEN had clear resection margin, while only 7 of the 9 patients (77.8%) with gallbladder NEN received R0 resection.

**Table 3:** Clinicopathological features of biliary neuroendocrine tumors after curative resection according to the World Health Organization 2010 classification.

	**G1 *n* = 1**	**G2 *n* = 3**	**G3 *n* = 18**	**MANEC *n* = 6**
Gender (male, %)	0	2 (66.7)	15 (83.3)	2 (33.3)
Median age (yr)	53	41.3	55.4	62
Median Ki-67 index (%)	1	3	60	70
Lymphovascular invasion (%)	1 (100)	1 (33.3)	11 (61.1)	4 (66.7)
**INITIAL METASTASIS (%)**				
Lymph node	1 (100)	1 (33.3)	10 (55.6)	4 (66.7)
Liver	0	0	2 (11.1)	1 (16.7)
Pancreas	0	2 (66.7)	3 (16.7)	0
**TNM STAGING (%)**				
0	0	1 (33.3)	1 (5.6)	0
I	0	0	5 (27.8)	0
II	1 (100)	1 (33.3)	7 (38.9)	6 (100)
III	0	1 (33.3)	4 (22.2)	0
IV	0	0	1 (5.6)	0
**RESECTION MARGIN (%)**				
R0	1 (100)	3 (100)	17 (94.4)	5 (83.3)
R1	0	0	1 (5.6)	1 (16.7)
Median primary tumor size (cm)	1.4	2.1	2.7	2.9
Primary tumor size>2cm (%)	0	1 (33.3)	9 (50)	5 (83.3)
Recurrence (%)	0	0	9 (50)	4 (66.7)
Median DFS (mo)	14	58	6	9.5
Median OS (mo)	14	58	25	23
2-Year survival rate (%)	N/A	100	55.6	66.7

*NEC, neuroendocrine carcinoma; MANEC, mixed adenoneuroendocrine carcinoma; DFS, disease free survival; OS, overall survival*.

### Therapy

Six patients with gallbladder NEN were diagnosed with gallbladder cancer pre-operation, thus underwent radical resection. While the other three patients were incidentally diagnosed as NEN according to pathology results after receiving laparoscopic cholecystectomy. One patient with bile duct NEN underwent radical resection of cholangiocarcinoma, while the other patient received cholecystectomy and cholangioenteric anastomosis. Fifteen patients with AoV NEN underwent pancreaticoduodenectomy. Two patients with AoV tumor only received resection of the tumor.

### Clinical Outcomes

**Figure 2:**
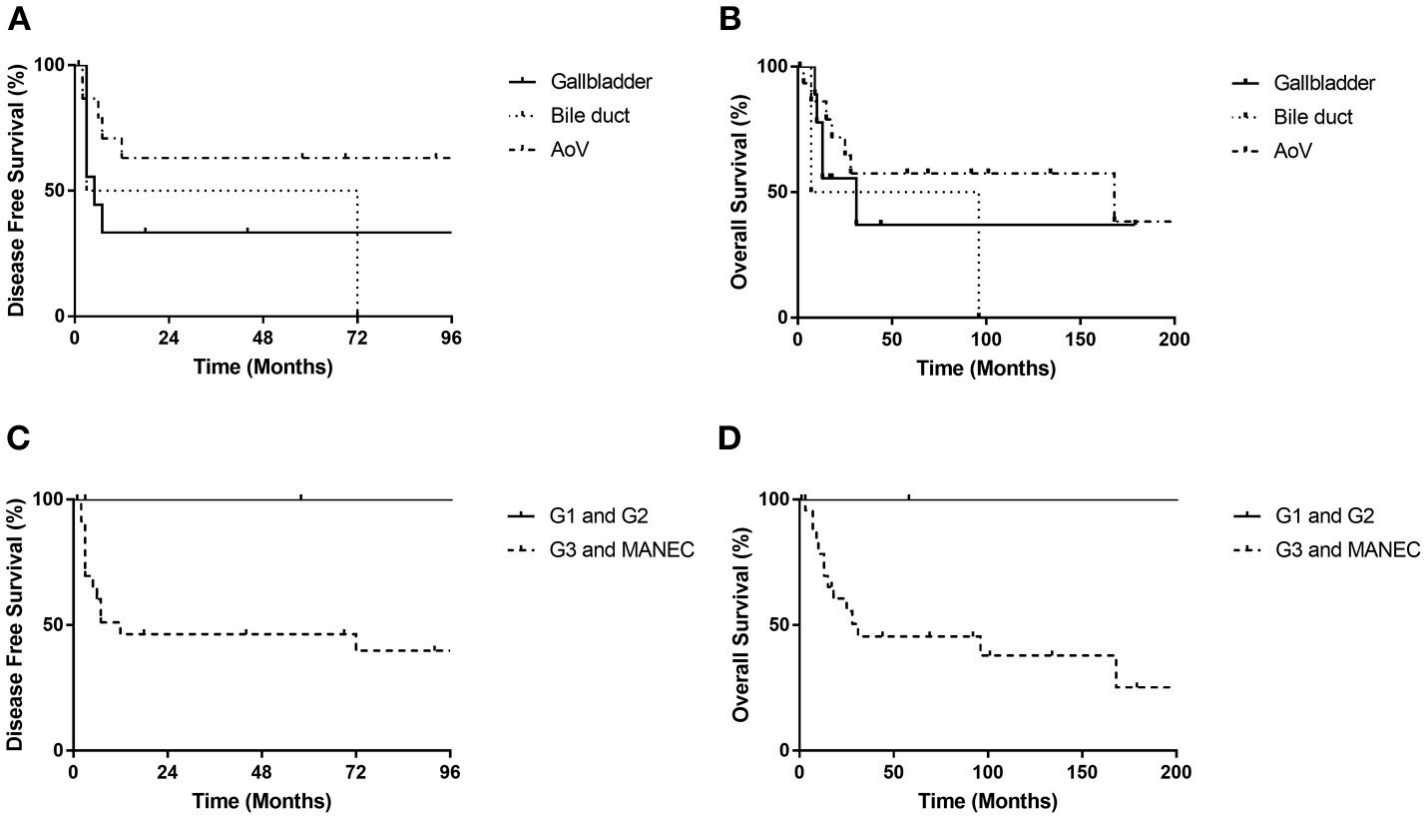
Disease free survival rates and overall survival rates of biliary NET patients. There were no significant differences in DFS or OS rates according to location of primary tumor (DFS, *p* = 0.185; OS, *p* = 0.401) **(A,B)** or the grading of tumors (DFS, *p* = 0.152; OS, *p* = 0.150) **(C,D)**. AoV, ampulla of Vater.

Disease-specific survival analysis observed 12 disease-related deaths, including five patients with tumor from the gallbladder (*n* = 9), two from the bile duct (*n* = 2) and five from the ampulla of Vater (*n* = 17) (Figure 2). There were no significant differences in the disease-free survival (DFS) or overall survival (OS) rates between NENs from different locations (*p* = 0.185 for DFS and *p* = 0.401 for OS). No disease-related deaths were observed in G1 and G2 subgroup. There were eight deaths in the G3 group (*n* = 18) and four deaths in the MANEC group (*n* = 6). However, neither the DFS rate nor OS rate had a significant difference between G1 and G2 NETs and NEC or MANEC groups (*p* = 0.152 for DFS, *p* = 0.150 for OS) (Figure 2). Also, there were no significant differences in the DFS rate or OS rate between NEC and MANEC groups (*p* = 0.715 for DFS and *p* = 0.670 for OS).

**Figure 3:**
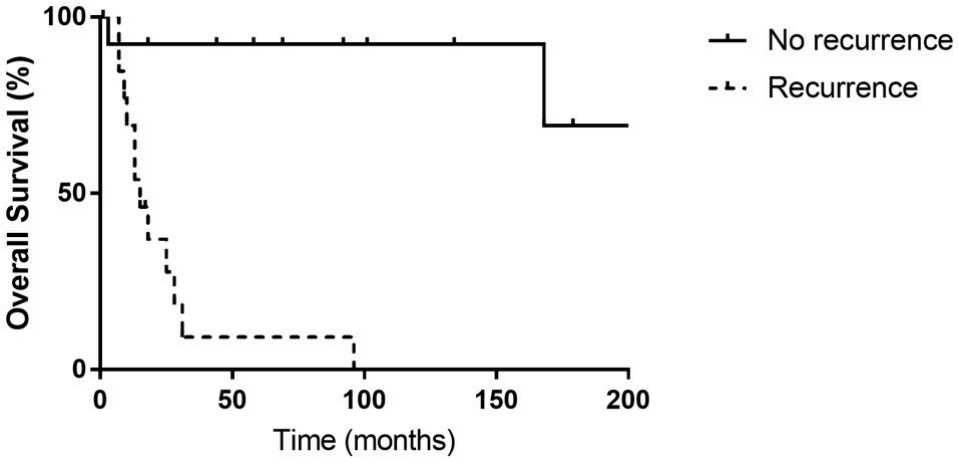
There was a significant difference between the overall survival rates of patients with or without recurrence (*p* < 0.001).

Patients that suffered from recurrence had statistically significant lower OS rates compared with patients without recurrence (*p* < 0.001) (Figure 3). We did not observe any recurrence for G1 and G2 NETs, while 54.2% of G3 and MANECs suffered recurrence (*p* > 0.05).

**Figure 4:**
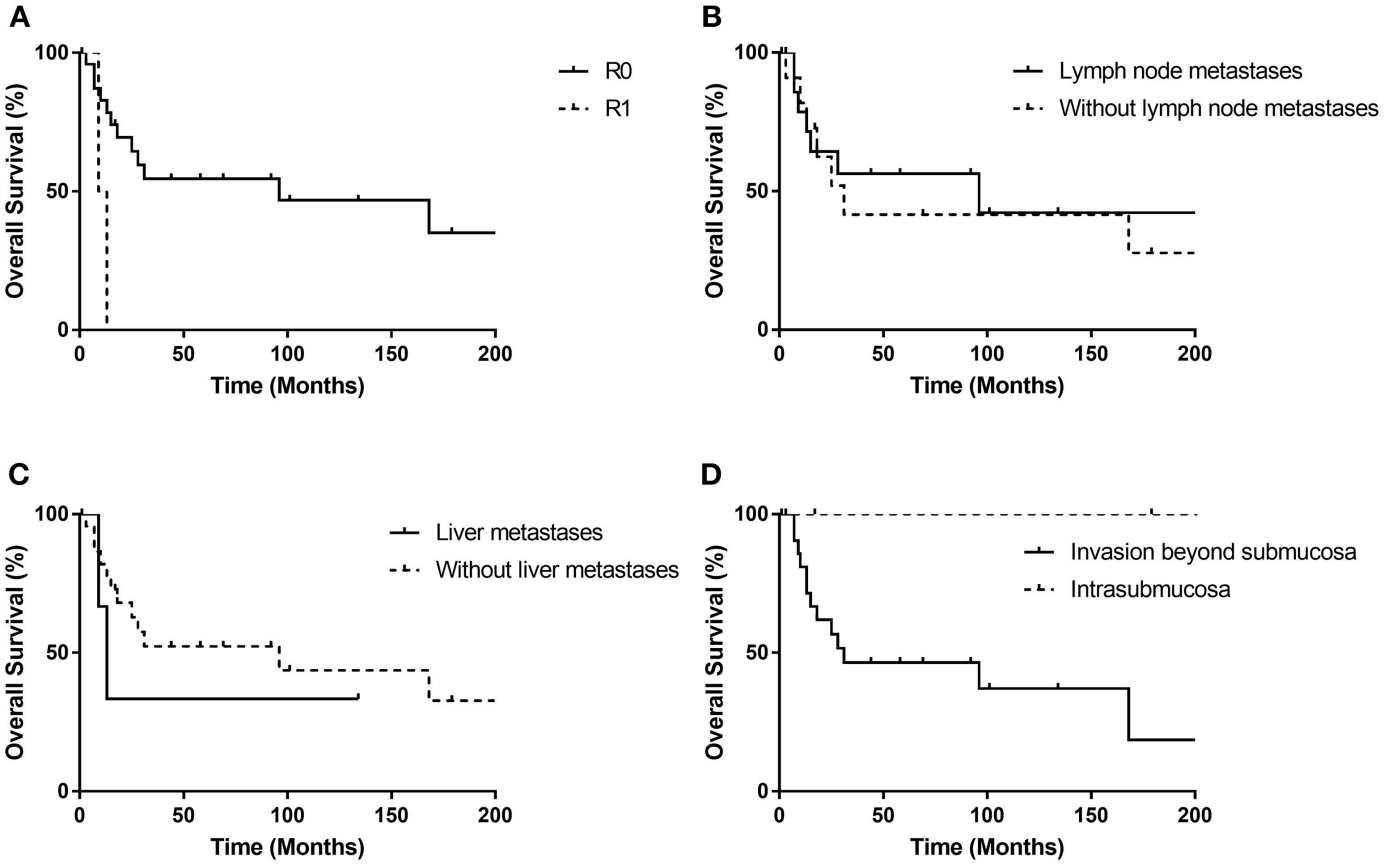
Kaplan-Meier curves for the overall survival stratified by possible prognostic factors **(A–D)**. R0 resection was related with longer OS (*p* = 0.021), while lymph node metastasis (*p* = 0.733), liver metastasis (*p* = 0.505) and invasion beyond submucosa (*p* = 0.076) did not show statistically significant correlations.

Figure 4 shows the Kaplan-Meier curves of overall survival for possible predictable factors of prognosis. Receiving R0 resection was related with longer OS (*p* = 0.021), while lymph node metastasis (*p* = 0.733), liver metastasis (*p* = 0.505) and invasion beyond submucosa (*p* = 0.076) did not show statistically significant correlations.

Table 4 shows that Ki-67 index>65% and tumor size>3.6 cm are possible cutoff values for prediction of local lymph node metastases. While, univariate analysis only showed that lymphovascular invasion (*p* < 0.001) and invasion beyond the submucosa (*p* = 0.024) were related to higher risk of local lymph node metastases (Table 5). Multivariate analysis identified a younger patient age [hazard ratio (HR), 1.069; 95% confidence intervals (CI), 1.010–1.132; *p* = 0.021] and R0 resection margin [HR, 6.886; 95% CI, 1.248–38.002; *p* = 0.027] as statistically significant prognostic factors associated with longer OS.

**Table 4:** Cutoff values for prediction of local lymph node metastases.

		**Sensitivity (95% CI)**	**Specificity (95% CI)**	**AUC ROC**
Ki-67	65%	37.5% (15.2–64.57)	75% (42.81–94.51)	0.5208 (0.293–0.7487)
Tumor size	3.6cm	37.5% (15.2–64.57)	91.67% (61.52–99.79)	0.6042 (0.389–0.8193)

*AUC, Area under the curve*.

**Table 5:** Variables associated with local lymph node metastases.

	**Odds ratio**	**95% CI**	***p*-value**
G3 vs. G2	2.8	0.2837–43.05	0.5692
Lymphovascular invasion	Infinity	18.62-Infinity	< 0.0001
Invasion beyond the submucosa	Infinity	1.385-Infinity	0.0242
Ki-67>65%	1.8	0.3393–7.944	0.6870

## Discussion

In this study, clinicopathological profiles of 28 neuroendocrine neoplasms originating from the gallbladder, the extrahepatic bile duct and the ampulla of Vater were identified. In accordance with previously reported case series (6), our study also showed that the ampulla of Vater was the most common location of biliary NENs. There were no cases of intrahepatic biliary NEN, and no cases of functioning biliary NEN as well. Detection of gallbladder NENs at an early stage was very difficult because patients lack characteristic symptoms. Fifty-eight percent of patients that had NEN in the AoV presented with jaundice, making this location the easiest for diagnosis. G1 and G2 NETs cases account for 14.2% among all NET cases, much less than the percentage (52.3%) reported from a SEER study (8), but close to the percentage (14.3%) reported in an Korean study (4), indicating a possible ethnical difference between Asian and Western populations.

For gallbladder cancers (GBC), the only curative treatment is radical cholecystectomy, but over 60% of GBC patients are not candidates for surgical resection at the time of diagnosis (12). However, gallbladder G1 and G2 NENs show less malignancy compared with GBC, thus surgical choices for gallbladder NETs vary. According to previous case reports, simple cholecystectomy has been used for T*in situ* or T1 gallbladder NENs, while aggressive radical surgery was recommended for high grade gallbladder NENs (13). In our study, six patients received radical cholecystectomy, including five R0 resections, and the median survival time reached 21.3 months. The other three patients were incidentally diagnosed with gallbladder NEN when receiving laparoscopic cholecystectomy, and none of them received a second radical surgery. The patient whose pathologic staging was T*in situ* has been disease-free until now without any adjuvant treatment. The patient whose pathologic staging was T2 was detected liver metastasis 3 months later. He received several courses of chemotherapy and transcatheter arterial chemoembolization, and was still alive now (17 months after surgery). The other patient whose pathologic staging was T3 was detected metastasis 3 months later and died 10 months after surgery. It has been recommended for incidentally detected GBC patients beyond T2 stage to receive a second radical surgery, but such guidelines are absent for gallbladder NENs. Our study showed that only for patients whom the gallbladder NEN is T*in situ*, simple cholecystectomy can be an adequate treatment.

NETs from the ampulla of Vater should be differentiated from another kind of benign tumor commonly arising from the second part of duodenum: gangliocytic paragangliomas (GPs). Due to several morphologic similarities, GP is often misdiagnosed as G1 NETs (14). And it has also been reported that GP accounts for ~40% of duodenal NETs (15). GPs show better prognosis compared with G1 NETs (16), thus it is important to differentiate GPs from NETs. Morphologically, GP comprises three characteristic cell types: epithelioid cells, spindle-shaped cells and ganglion-like cells. Previous studies found that the epithelioid cells from GP exhibit a positive immunoexpression for pancreatic polypeptide and progesterone receptor, while G1 NETs are stained negative (17). In our study, the tumor samples from the ampulla of Vater were all carefully stained for pancreatic polypeptide to exclude GPs.

Multivariate analysis indicated that patient age and receiving R0 resection were related with better prognosis. Consistent with previous studies, our multivariate analysis did not indicate any difference in survival between tumors at different locations (4). In a Korean study, Kim et al found out that NET graded as G1 and G2 was associated with a better prognosis (4). And another study showed that G3 was the independent prognostic factor associated with overall survival (6). However, our study did not show a correlation between tumor grading and prognosis, although, the 2-year survival rate for G1 and G2 NENs in our study was 100%, while for G3 NECs and MANECs, 2-year survival rate was only 55.6 and 66.7%, respectively. The limited sample size may have prevented us from showing the association in our study, as our G1 and G2 group only included four patients.

Our study had some limitations, such as imperfect data collection and a rather short follow-up time for some patients. Also, the number of patients was not enough to conduct an effective multivariate analysis. However, this study still included relatively large numbers of biliary tract NENs with intact follow-up information. For such a rare group of neuroendocrine tumor, we also call on for a local registry or database, which would be helpful in further understanding and management of these patients.

## Conclusions

Neuroendocrine neoplasm has been an emerging disease in recent years, but biliary NENs still appear to be extremely rare. Our study was the first single center study in China that included the greatest number of cases of biliary NENs. This study summarized the clinical characteristics, management and prognosis profiles of 28 biliary NEN patients from a large tertiary center. No hormonal symptoms were observed, and the lack of characteristic symptoms makes it difficult for early diagnosis. Recurrence of disease correlated with poor prognosis. Lymphovascular invasion and invasion beyond the submucosa were related to higher risk of local lymph node metastases. Multivariate analysis identified patient age and R0 resection margin as independent prognostic factors associated with overall survival. Future studies based on multicenter database or national registries are needed for such a rare group of neuroendocrine tumors.

## Author Contributions

ZZ, CC, BL, CZ, and YZ: conceptualization; CZ, XH, WL, and TH: data curation; BL: Funding acquisition; ZZ, CC, BL, and HL: methodology; ZZ and CC: software; BL: supervision; LZ and HZ: validation; ZZ and CC: writing original draft; BL, HL, LZ, HZ, CZ, XH, WL, TH, and YZ: writing review and editing.

### Conflict of Interest Statement

The authors declare that the research was conducted in the absence of any commercial or financial relationships that could be construed as a potential conflict of interest.
